# Immunogens Modeling a Fusion-Intermediate Conformation of gp41 Elicit Antibodies to the Membrane Proximal External Region of the HIV Envelope Glycoprotein

**DOI:** 10.1371/journal.pone.0128562

**Published:** 2015-06-18

**Authors:** Russell Vassell, Yong He, Prasad Vennakalanti, Antu K. Dey, Min Zhuang, Wei Wang, Yide Sun, Zohar Biron-Sorek, Indresh K. Srivastava, Celia C. LaBranche, David C. Montefiori, Susan W. Barnett, Carol D. Weiss

**Affiliations:** 1 Center for Biologics Evaluation and Research, Food and Drug Administration, Silver Spring, Maryland, United States of America; 2 Novartis Vaccines and Diagnostics, Cambridge, Massachusetts, United States of America; 3 Department of Microbiology, Harbin Medical University, Heilongjiang, China; 4 Department of Surgery, Duke Medical Center, Durham, North Carolina, United States of America; Shanghai Medical College, Fudan University, CHINA

## Abstract

The membrane proximal external region (MPER) of the gp41 subunit of the HIV-1 envelope glycoprotein (Env) contains determinants for broadly neutralizing antibodies and has remained an important focus of vaccine design. However, creating an immunogen that elicits broadly neutralizing antibodies to this region has proven difficult in part due to the relative inaccessibility of the MPER in the native conformation of Env. Here, we describe the antigenicity and immunogenicity of a panel of oligomeric gp41 immunogens designed to model a fusion-intermediate conformation of Env in order to enhance MPER exposure in a relevant conformation. The immunogens contain segments of the gp41 N- and C-heptad repeats to mimic a trapped intermediate, followed by the MPER, with variations that include different N-heptad lengths, insertion of extra epitopes, and varying C-termini. These well-characterized immunogens were evaluated in two different immunization protocols involving gp41 and gp140 proteins, gp41 and gp160 DNA primes, and different immunization schedules and adjuvants. We found that the immunogens designed to reduce extension of helical structure into the MPER elicited the highest MPER antibody binding titers, but these antibodies lacked neutralizing activity. The gp41 protein immunogens also elicited higher MPER titers than the gp140 protein immunogen. In prime-boost studies, the best MPER responses were seen in the groups that received DNA priming with gp41 vectors followed by gp41 protein boosts. Finally, although titers to the entire protein immunogen were similar in the two immunization protocols, MPER-specific titers differed, suggesting that the immunization route, schedule, dose, or adjuvant may differentially influence MPER immunogenicity. These findings inform the design of future MPER immunogens and immunization protocols.

## Introduction

Efforts to generate a broadly protective vaccine for HIV/AIDS have been thwarted by the inability to create immunogens that elicit neutralizing antibodies to conserved sites in the envelope glycoprotein (Env). Conserved neutralizing determinants in Env are shielded from antibodies by various mechanisms, including extensive glycosylation and conformational masking that limit antibody access to neutralizing sites [1,2]. Some conserved neutralizing sites, such as the co-receptor binding site in gp120 and heptad-repeat regions in gp41, only become transiently exposed as Env transitions through a series of conformational changes triggered by receptor binding and leading to membrane fusion [3–11]. Nonetheless, broadly neutralizing antibodies to various sites can be found in some persons infected with HIV, but titers are generally low and only emerge after several years of infection [12–17].

Until relatively recently only a limited number of broadly neutralizing monoclonal antibodies had been isolated. These monoclonals identified three key neutralizing determinants, namely the CD4 binding site, a glycan moiety on gp120, and the membrane proximal external region (MPER) [18–23]. More recently, new high-throughput technologies have facilitated the identification of many more potent, broadly neutralizing monoclonal antibodies [24–32]. Many of these new monoclonals recognize conformational neutralizing determinants in V2 and V2-V3, sometimes involving glycan moieties. These newly identified neutralizing determinants, along with the ones identified previously, have been the focus of intense investigations involving wide-ranging approaches to design vaccines that can elicit antibodies to these conserved sites [33,34].

High-resolution structural studies have further refined our understanding of features of the broadly neutralizing antibody paratopes and neutralizing epitopes [31,35–42]. Such studies have aided the design of novel immunogens that precisely mimic neutralizing epitope structures in the antibody-bound conformation [43,44], often involving transplanting epitopes onto unrelated protein scaffolds for improving epitope accessibility and stability [45–48]. The recent high-resolution structures of a stabilized, pre-fusion Env trimer have further shed light on constraints to antibody access [49–51].

Recent studies characterizing the genetic evolution of broadly neutralizing antibodies have also provided insights into changes in antibody binding to its cognate Env determinant during antibody maturation [52]. Accumulation of a large number of somatic mutations as the antibody acquires increasing affinity for a broader range of Envs has been seen for many monoclonals, including the 2F5 monoclonal antibody that targets the MPER [53,54]. These findings raise intriguing questions about the nature of the antigens that trigger development of an antibody along a particular genetic pathway. For example, does the evolution of the HIV quasispecies during natural infection play an important role in driving antibody maturation pathways to more conserved sites in Env? Additionally, it has been proposed that the poor immunogenicity of the MPER relates to immune tolerance [55].

To overcome HIV diversity, vaccines may need to elicit neutralizing antibodies to several sites on Env to increase the likelihood that at least one neutralizing determinant in Env will be vulnerable. Therefore, efforts to design immunogens that enhance responses to each of the known neutralizing determinants must continue. The MPER remains an attractive target for neutralizing antibodies because it is highly conserved and broadly neutralizing antibodies are elicited to this region in some infected persons. Many strategies have been employed over the years to develop MPER-directed vaccines, including the display of MPER epitopes in membranes, virus-like particles, chimeric viral antigens, and in a variety of gp140 and gp41 constructs [56–67]. Modest gains in neutralization breadth or potency have been achieved with some of these approaches, but continued development of better MPER-based vaccines is needed.

Here, we describe our efforts to focus antibody responses to the poorly immunogenic MPER by designing novel, oligomeric gp41 immunogens that model a fusion-intermediate conformation of Env. Prior studies of receptor-induced conformational changes in Env indicate that fusion intermediate-type conformations may better expose the MPER relative to the native pre-fusion conformation or the final, six-helix bundle (6HB) post-fusion conformation of Env [68–73]. Accordingly, we created a panel of well-characterized gp41 immunogens that lack gp120 to eliminate its contribution to epitope masking, but retain trimeric structure due to self-assembly of N- and C-terminal, heptad-repeat segments (N-HR and C-HR, respectively) into a partial 6HB (short 6HB)adjacent to the MPER. Our strategy differs from other MPER-directed gp41 immunogens that use only C-HR or MPER segments or additionally include longer N-HR segments that are capable of assembling with the C-HR to form the complete gp41 core (long 6HB) [56,62,63,74,75]. We analyzed several variations of gp41 trimers in protein only and prime-boost protocols involving gp41 and gp160 DNA primes and gp41 and gp140 protein boosts. Our studies show that the gp41 protein immunogens in our panel that were designed to mitigate extension of helical structure in MPER, which may more closely resemble a fusion-intermediate conformation of gp41, elicited the highest antibody binding titers to the MPER, though these antibodies lacked significant neutralizing activity. We further describe how various modifications in the gp41 immunogens or immunization protocol, such as inclusion of a V3 immunodominant epitope in the protein antigen or use of gp160 or gp41 DNA priming, affected antibody responses to the MPER. These findings provide new information for designing next-generation MPER immunogens and immunization protocols.

## Materials and Methods

### Ethics Statement

The animal studies were conducted according to protocols approved by the Institutional Animal Care and Use Committees at CBER, FDA and Novartis Vaccines and Diagnostics, in accordance with the requirements for the humane care and use of animals as set forth in the Animal Welfare Act, the Institute for Laboratory Animal Research Guide for the Care and Use of Laboratory Animals, and all applicable local, state, and federal laws and regulations. Single immunogen studies were undertaken at the CBER under the direction of CBER Division of Veterinary Services (ASP No. 1998–02). Prime-boost studies were undertaken at Josman LLC (Napa, CA), a research facility licensed through the USDA (No. 93-R-0260) with Public Health Service Assurance from the NIH (No. A3404-01).

### Reagents

2F5 and 4E10 monoclonal antibodies were purchased from Polymun Scientific (Vienna, Austria). NC-1 monoclonal antibody [76] was kindly provided by Shibo Jiang (New York Blood Center). The Chessie 8 monoclonal gp41 antibody [77] was obtained through the AIDS Reagent Program, Division of AIDS, NIAID, NIH from George Lewis (Institute of Human Virology, University of Maryland). Epitope-scaffold antigens ES5-TH-Avi (referred to as ES5) and ES2-L (referred to as ES2) [44] were kindly provided by Peter Kwong (Vaccine Research Center, National Institutes of Health). The MPER peptide (NEQELLELDKWASLWNWFNITNWLWYIK), T20 peptide (YTSLIHSLIEESQNQQEKNEQELLELDKWASLWNWF), C34 peptide (WMEWDREINNYTSLIHSLIEESQNQQEKNEQELL) and N36 peptide (SGIVQQQNNLLRAIEAQQHLLQLTVWGIKQLQARIL) were made by standard 9-fluorenylmethoxy carbonyl chemistry and purified by HPLC (CBER Facility for Biotechnology Resources, Food and Drug Administration) and confirmed to have the expected molecular weight by matrix-assisted laser desorption ionization-time-of-flight mass spectroscopy. The expression plasmid for N34C28 [78] was provided by Min Lu (Weill Medical College of Cornell University), and this protein was expressed and purified in the same manner as the immunogens described below.

### Immunogen expression and purification

**Fig 1:**
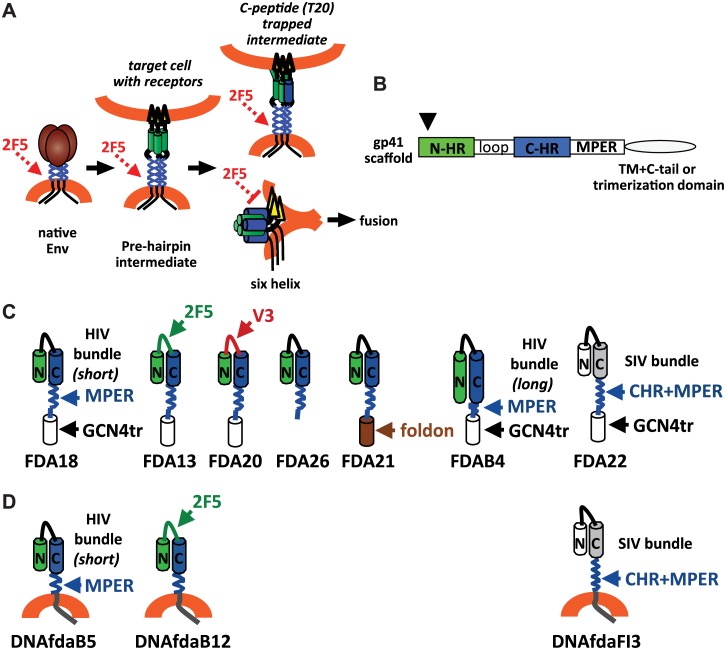
gp41 conformational changes and gp41 immunogens. (A) Diagram of the envelope glycoprotein trimer undergoing receptor-induced conformational changes, transitioning from native, to pre-hairpin fusion intermediate, to the final six-helix bundle conformation. The 2F5 monoclonal antibody binds native and pre-hairpin intermediate, but not six-helix conformations of gp41. Trapping the prehairpin intermediate with a peptide corresponding to the C-HR preserves 2F5 binding after receptor-induced conformational changes. (B) gp41 domains used in the various immunogens. Arrow indicates truncation of the N-terminus of the N heptad repeat (N-HR) at residue 553 (HXB2 numbering) used in in the immunogens with the short bundle (FDA18, FDA13, FDA20, FDA26, and FDA21). C-HR is the C-terminal heptad repeat. TM is the transmembrane domain. C-tail is the cytoplasmic domain. (C) Schematics of the protein immunogens used in this study, shown as a monomer for simplicity. A GCN4 trimerization domain is included in all immunogens except FDA21 and FDA26, which have a foldon trimerization or no trimerization domain, respectively. (D) Schematics of the protein expressed by the DNA priming vectors, shown as monomers for simplicity.

Plasmids expressing the gp41 constructs shown in Fig 1C were created by PCR amplification of gp41 sequences from the HXB2 *env* gene from the pSM WT expression vector [79], and, in the case of the FDA22 immunogen, the SIV_mac239_ *env* gene from the pET11a expression vector [80], followed by addition of C-terminal or loop extensions using standard molecular biological techniques. Protein sequences for the gp41 constructs are listed in S1 Table. The gp41 constructs were cloned into the pET25b(+) vector (Novagen), confirmed by sequencing, and expressed in *E*. *coli* BL21(DE3) (Novagen). The gp41 recombinant proteins were then isolated from inclusion bodies, purified by high performance liquid chromatography (HPLC) using Delta PAK-C18 columns (Waters), and refolded by dialyzing sequentially against serial 20 mM Tris (pH8.0) buffers supplemented respectively with 6.0, 3.0,1.0, and 0.5 M guanidine hydrochloride for 4 hours at 4°C for each step. The final renatured protein solutions were dialyzed in phosphate-buffered saline in order to remove the denaturing agents. All recombinant proteins were confirmed to have the expected molecular weight by matrix-assisted laser desorption ionization-time-of-flight mass spectroscopy and assessed to be at least 95% pure by gel electrophoresis (10% NuPage Gel, Life Technologies) and staining with GelCode Blue (Pierce). Recombinant gp140 oligomers (SF162 strain) used in prime-boost studies were generated in mammalian cells and characterized as previously described [81,82].

### Immunogen characterization

Oligomerization of the purified gp41 proteins was analyzed by size exclusion HPLC using BioSep-SEC- S3000 (Phenomenex) or Zorbax GF250 columns (Agilent Technologies). To assess helical content and thermal stability as indicators of potential 6HB structure, the immunogens were additionally analyzed by circular dichroism (CD) spectroscopy, as described previously [83]. Briefly, CD spectra of the recombinant proteins were acquired on a Jasco spectropolarimeter (model J-810, Jasco, Inc.) at room temperature using a 1.0 nm bandwidth, 0.1 nm resolution, 0.1 cm path length, 4.0 seconds response time, and a 5-nm/min scanning speed. Thermal denaturation was monitored at 222 nm by applying a thermal gradient of 2°C/min in the range of 4–95°C. Reverse melt from 95°C to 4°C was also detected. The melting curve was smoothed, and the midpoint of the thermal unfolding transition (T_m_) value was determined using Jasco software. The T_m_ averages of at least two measurements for each complex were calculated.

Surface Plasmon Resonance (SPR)-based BIAcore 3000 was used to asses binding affinities of the 2F5 and 4E10 monoclonal antibodies to the soluble immunogens (Table 1). Briefly, approximately 200 RU of 2F5 or 4E10 monoclonal antibodies were immobilized directly onto a CM5 sensor chip via amine coupling. Varying concentrations of the immunogens were then injected at 80 μl/min. The binding analyses were performed at 25°C with HBS-EP running buffer. The experimental curves were fitted to a 1:1 Langmuir binding model using BIAevaluation software 3.2 (BIAcore Inc). The association rate, K_on_, dissociation rate, K_off_, and the equilibrium dissociation constant, K_D_, are derived following the 1:1 fit.

**Table 1:** SPR analysis of monoclonal antibody binding to gp41 oligomers.

Immunogen (antibody)	K_on_ (1/Ms)	K_off_ (1/s)	K_D_ (M)
**FDA18 (2F5)**	4.73e3	8.34e-5	**1.76e-8**
**FDA18 (4E10)**	3.9e3	8.24e-4	**2.12e-7**
**FDA13 (2F5)**	1.35e5	6.69e-5	**4.95e-10**
**FDA13 (4E10)**	1.17e3	6.11e-4	**5.24e-7**
**FDA20 (2F5)**	9.67e3	9.21e-4	**9.52e-8**
**FDA20 (4E10)**	1.18e3	1.04e-4	**8.81e-8**
**FDA26 (2F5)**	2.31e4	9.03e-4	**3.91e-8**
**FDA26 (4E10)**	1.44e4	1.95e-3	**1.35e-7**
**FDA21 (2F5)**	2.26e4	4.2e-4	**1.86e-8**
**FDA21 (4E10)**	1.87e4	4.57e-3	**2.44e-7**
**FDAB4 (2F5)**	1.35e4	6.37e-4	**4.73e-8**
**FDAB4 (4E10)**	7.47e3	2.33e-3	**3.12e-7**
**FDA22 (2F5)**	2.19e4	3.56e-4	**1.68e-8**
**FDA22 (4E10)**	6.54e3	5.28e-4	**8.08e-8**

### DNA priming vectors

Three eukaryotic expression plasmids called fdaB5, fdaB12, and fdaFI3 were derived from gp41 sequences in the expression plasmids for FDA18, FDA13, and FDA22, respectively. Gp41 sequences from the FDA immunogen constructs, without the C-terminal heterologous sequences used for trimerization, were PCR amplified and joined to sequences coding for the transmembrane and cytoplasmic tail regions of gp41 from the HXB2 strain during cloning into the pCMVlink eukaryotic expression vector [84]. All plasmids were sequenced to confirm the presence of intended sequences. Expression of the desired constructs was verified in transfected cell lysates by SDS-PAGE (Novagen), followed by immunoblotting with the Chessie 8 monoclonal gp41 antibody [77] and detection by chemiluminescence (KPL).

### Immunizations

Initial immunogenicity studies involving only protein immunogens were undertaken at CBER, FDA Division of Veterinary Services. Three NZW female rabbits per group for FDA13, FDA18, FDA22, and FDAB4, and two rabbits per group for FDA20 and FDA21 immunogens, were primed with a single dose of 50 μg of protein mixed with an equal volume of complete Freund’s adjuvant, delivered subcutaneously at two sites. Three boosts of 50 μg protein mixed with an equal volume of incomplete Freund’s adjuvant were similarly administered at approximately four week intervals. In addition, one group of six rabbits received sequential immunizations with two doses each of FDA13, FDA18, and FDA22 given at four week intervals. For this group, two rabbits each were immunized with one of the following regimens: 1) FDA13, FDA18, FDA22, FDA13, FDA22, and FDA18; 2) FDA13, FDA22, FDA18, FDA13, FDA22, and FDA18; or 3) FDA18, FDA13, FDA22, FDA13, FDA22, and FDA18.

In the prime-boost studies, five NZW female rabbits per group for studies #1–3 and four NZW rabbits per group for study #4 were primed at weeks 0 and 4 with either 1 mg of DNA-vectored immunogen delivered IM at four sites or 25 μg of protein immunogen mixed with MF59 adjuvant delivered IM at two sites. Subsequent boosts with 25 μg of protein immunogen mixed with MF59 adjuvant administered IM in two sites were given at weeks 12 and 24.

At the completion of experiments, rabbits were exsanguinated under Ketamine and Xylazine anesthesia followed by asphyxiation in a dedicated C0_2_ chamber.

### ELISA assays

The presence of a 6HB structure was further assessed in an ELISA using the NC-1 monoclonal antibody that is specific for the 6HB [76]. Briefly, 96-well polystyrene plates (Immulon II HB, Thermo Scientific) were coated with 50 μl/well of a 1.0 μM solution of the various recombinant proteins in PBS and incubated overnight at RT. Unbound protein was removed and plates were blocked with 300 μl /well of 5% nonfat dry milk in wash buffer (TBS-T; 150 mM NaCl, 20 mM TRIS pH 8.0, and 0.05% Tween-20). After removing blocking buffer, serial dilutions of NC-1 culture supernatant from 1:25 to 1:18225 diluted in blocking buffer were applied at 50 μl /well and incubated 1 hour at 37°C. Plates were washed and 50 μl /well of peroxidase labeled goat anti-mouse-IgG (H+L) (KPL) was applied and incubated 30 minutes at room temperature. Plates were washed prior to adding 100 μl /well of SureBlue TMB microwell peroxidase substrate (KPL) and incubating 20–30 min at room temperature. The reaction was stopped by adding 100 μl /well of 1M sulfuric acid, and plates were read at 450nm on an absorbance plate reader. Titers are expressed as ELISA units (EU), defined by multiplying the highest dilution factor of sera giving a signal above background (OD_450nm_ > 0.2) by the OD_450nm_ at that dilution.

Antibody responses to the immunogen or MPER determinants were assessed in ELISAs, as described above except that immunogens, peptides, or 2F5 epitope scaffold proteins were coated onto the plates and probed with serial dilutions of rabbit sera followed by HRP labeled goat anti-rabbit. For data shown in Tables 2 and 3, rabbit sera were pooled by group prior to performing the assay. Data shown are mean values from two independent ELISAs. For data comparing the 2F5 scaffold proteins and the MPER peptide, individual rabbits were assayed, and group averages are shown. Data points shown are means values from two independent ELISAs. A one-sided *t* test was applied to determine the statistical significance between the groups.

**Table 2:** ELISA titers to the indicated antigens.

Group	Immunogen	MPER titer* (s.e.m.)	T20 titer* (s.e.m.)	Immunogen titer* (s.e.m.)	MPER/Immu-nogen titer (%)
S2	FDA18	3695** (689)	8095 (1754)	74,379 (837)	5.0
S1	FDA13	3065** (1029)	7605 (1519)	60,404 (1362)	5.1
S3	FDA20	460 (74)	3058 (296)	56,204 (2462)	0.8
S6	FDA26	5213** (801)	6818 (1326)	39,579 (4137)	13.2
S4	FDA21	990** (176)	3533 (776)	45,504 (2538)	2.2
S7	FDAB4	80 (13)	740 (166)	63,029 (537)	0.1
S5	FDA22	318 (18)	1780 (995)	81,754 (4162)	0.4
H1	FDA13, 18, 22 ***	2364** (66)	5415 (1194)	59321 (2046)	4.0

* titer = mean ELISA units (EU, endpoint dilution x O.D. _450nm_) from two independent assays. (s.e.m.) = standard error of mean

**Indicates significantly higher than FDAB4 by one-sided *t* test.

*** Two rabbits each were immunized with two doses each of the indicated antigens given in various order as described in Materials and Methods.

**Table 3:** ELISA titers to the indicated antigens.

Group	Immunogen prime/boost*	MPER titer** (s.e.m.)	Immunogen titer** (s.e.m.)
**Prime-boost study #1**			
N1-1	gp140/gp140	n.d.	2190***
N1-2	FDAB4/FDAB4	46 (16)	37,954 (588)
N1-3	gp140/FDAB4	n.d.	6830*** (139)
N1-4	FDAB4/gp140	28 (8)	5655*** (116)
N1-7	DNA gp160/ gp140	n.d.	2370*** (116)
N1-8	DNA gp160/FDAB4	n.d.	28,304***(488)
**Prime-boost study #2**			
N2-1	FDA13/FDA13	46 (21)	40,679(46)
N2-2	FDA18/FDA18	23 (12)	42,579 (4363)
N2-3	DNAgp160/FDA18	44 (12)	31,129 (1337)
**N2-4**	**DNAfdaB12/FDA18**	**101 (23)**	32,429 (513)
N2-5	FDA13/gp140	n.d.	4835 (99)
N2-6	FDA18/gp140	n.d.	4515 (171)
**N2-7**	**DNAfdaB12/FDA13**	**526 (162)**	38,304 (1938)
N2-8	DNAfdaB12/gp140	n.d.	681 (23)
N2-9	DNAgp160/FDA13	84 (29)	34,654 (4138)
**Prime-boost study #3**			
N3-1	DNAfdaFI3/FDA22	64 (20)	52,529 (63)
N3-2	DNA fdaB5/FDA22	35 (4)	56,754 (3412)
N3-3	DNAgp160/FDA22	n.d.	51,204 (1712)
N3-4	DNAfdaFI3/FDA18	n.d.	38,254 (312)
N3-5	DNAfdaB5/FDA18	24 (7)	38,979 (3137)
N3-6	DNAgp160/FDA18	n.d.	32,954 (2538)
**N3-7**	**DNA fdaFI3/FDA20**	**548 (107)**	79,054 (938)
N3-8	DNAfdaB5/FDA20	51 (16)	64,079 (1537)
N3-9	DNAgp160/FDA20	n.d.	68, 354 (388)
N3-10	FDA22/FDA22	n.d.	48,579 (2937)
N3-11	FDA18/FDA18	35 (7)	42,679 (2987)
N3-12	FDA20/FDA20	n.d.	50,479 (1013)
**Prime-boost study #4**			
N4-1	DNAfdaB12+FDA26/FDA26	42 (17)	23,954 (2238)
N4-2	FDA26/FDA26	n.d.	24,254 (588)

*Two primes giving at weeks 0 and 8 followed by boosts at weeks 12 and 24 using the indicated immunogens mixed with MF59 adjuvant.

** titer = mean ELISA unit (EU, endpoint dilution x O.D. _450nm_) to the MPER peptide or protein immunogen using sera following the final boost.

*** tested against FDAB4 immunogen. s.e.m. = standard deviation; n.d. = not detectable above background

### Pseudovirus Neutralization Assays

Sera for all individual rabbits in the protein only study, and pooled sera from all groups in all prime-boost studies were assessed for neutralization of SF162 and HXB2 in a pseudotype neutralization assay using U87-CD4+CCR5+ cells for SF162 or U87-CD4+CXCR4+ cells for HXB2, as previously described [83]. Briefly, 0.5 μg of the Env in expression vector pCMV/R [85], 4–5 μg of the Env-deficient viral vector (pCMVΔ8.2) [86] and 4–5 μg of reporter vector (pHR’-Luc) [87] were co-transfected in 293T cells, and filtered supernatants of pseudovirus stocks were collected at 48 h posttransfection and stored at -80°C. pCMV/R, pCMVΔ8.2, and pHR’-Luc plasmids were kindly provided by Gary Nabel (VRC/NIH). Sera were pretreated at 56°C to inactivate complement prior to use. Pseudovirus stocks were pre-incubated with serial dilutions of sera at 37°C for 60 minutes prior to inoculation of 2×10^4^ cells/well in 96-well plates. Forty eight hours after infection, the cells were lysed, and luciferase activity was measured using Luciferase Assay System substrate according to manufacturer’s protocols (Promega) and a luminometer (L-max, Molecular Devices). Neutralization curves were generated using SoftMaxPro software (Molecular Devices). To assess contributions of antibodies directed to the V3 region for neutralization activity against SF162, a V3 peptide (EINCTRPNNNTRKSIRIGPGQAFYATGEIIGDIRQAHCNI) corresponding to the sequence used in the FDA20 immunogen, was added to the serum dilutions and incubated for 60 minutes at 37C to absorb V3-directed antibodies prior to mixing sera with virus and inoculating target cells, as described above. The V3 peptide was synthesized at CBER Facility for Biotechnology Resources, as described above.

Additional neutralization studies using sera from individual rabbits in all studies were performed in TZM-bl cells by the David Montefiori lab (Duke University) according to previously published protocols [88,89]. Neutralization of HIV-1 strains MN.3, W61D-TCLA, SF162.LS, WITO4160.3, 6535.3, QH0692.42, RHPA42259.7, and WITO4160.33 were assessed in TZM-bl cells. HIV-1 strains MN.3 and W62D-TCLA were used to evaluate sera from the protein only and prime-boost study #4. HIV-1 strains MN.3, W61D-TCLA, 6535.3, QH0692.42, RHPA42259.7 and WITO4160.3 were used to assess sera from prime-boost studies #1. HIV-1 strains W61D-TCLA, SF162.LS, and WITO4160.33 were used to assess sera from prime boost studies #2 and #3.

## Results

### Immunogen design

In our prior studies of Env conformational changes we observed that binding of the 2F5 antibody to gp41 is reduced after Env is triggered by soluble CD4 (sCD4) to form a 6HB [69]. Similar findings have also been observed by others [90] [91]. This result suggested that extension of helical structure from the 6HB core into the C-terminal end of the HR2 and MPER disrupts the 2F5 epitope (Fig 1A) and is supported by high-resolution structures showing the 2F5 epitope in non-helical conformations [35,37]. We further found that trapping the pre-hairpin fusion intermediate with a C-heptad peptide that lacked the 2F5 epitope (C34) abrogated the loss of 2F5 antibody binding to gp41 after treatment with sCD4 [69] (Fig 1A). This latter observation led us to design immunogens that resemble the peptide-trapped, pre-hairpin fusion intermediate of gp41, in order to better expose the 2F5 determinant and MPER.

Our main strategy for mimicking the peptide-trapped conformation of gp41 relied on use of a truncated (short) 6HB that would stabilize trimeric gp41 structure while also reducing potential helical extension of the C-HR into the 2F5 epitope and MPER. Based on our prior studies showing that an N-HR peptide (DP-107, residues 553–590 HXB2 numbering) can immunoprecipitate Env after sCD4 treatment and trap the fusion intermediate [11], we designed a gp41 immunogen that eliminates seven residues in the N-terminus of the N-HR. This construct creates a shorter N-HR that should still bind to the N-terminal region of the C-HR to form a short 6HB structure (Fig 1B, arrow). The gp41 immunogens also lack gp120 to avoid potential steric masking of gp41, and gp41 sequences N-terminal to the N-HR were excluded to avoid excessive hydrophobicity and potential interactions with downstream regions of gp41 [92] (Fig 1B and 1C, immunogens FDA18, FDA13, FDA20, and FDA26). In addition, the gp41 immunodominant loop between the N-HR and C-HR was replaced with a flexible linker, and the transmembrane domain and cytoplasmic tail were replaced with a GCN4 trimerization domain for immunogens FDA18, FDA13, FDA20 or a foldon trimerization domain for immunogen FDA21 (Fig 1C). These unrelated C-terminal extensions should further stabilize trimeric structure and were intended to approximate the effect of membrane anchoring. The trimer extensions are joined to the MPER by an eight-residue flexible linker to minimize potential structural constraints imposed by the helical trimerization domains. The FDA26 immunogen lacks a C-terminal trimerization domain and ends after the MPER (Fig 1C).

Other variations of the gp41 scaffold involve insertion of extra epitopes in the linker between the heptad repeats in order to enhance immunogenicity of the inserted epitope by placing it in a highly exposed loop in a trimeric format (Fig 1C). For the FDA13 immunogen, an extra 2F5 epitope was placed in the loop to assess whether this presentation would improve MPER focusing or affect elicitation of neutralizing antibodies directed to the MPER. For the FDA20 immunogen, a V3 sequence (clade C) was inserted in the loop to assess whether the presence of an immunodominant epitope would affect immunogenicity of the MPER and to evaluate the potential of the gp41 scaffold for presenting heterologous epitopes in a highly-exposed, trimeric format.

We also made an immunogen that includes seven additional N-terminal residues of the N-HR repeat that should allow formation of a more complete gp41 core (long) 6HB (FDAB4) (Fig 1C), but may impose helical structure on the MPER determinant and abrogate the 2F5 epitope structure. Finally, in an attempt to maximize exposure of the full C-HR, as well as the MPER, we also created the FDA22 immunogen that uses the SIV N-HR and C-HR for trimerization (Fig 1C). In this case, the SIV C-HR is joined to the complete HIV C-HR, MPER, and the linker-GCN4 trimerization domain. The greater stability of the SIV 6HB formed by the cognate SIV N-HR and C-HR likely favors formation of the SIV 6HB over a 6HB formed between the SIV N-HR and HIV C-HR, thus leaving the HIV C-HR and MPER exposed. Amino acid sequences for all of the protein immunogens are shown in S1 Table.

Three DNA priming vectors, DNAfdaB5, DNAfdaB12, DNAfdaFI3, which correspond to the protein immunogens FDA18, FDA13, and FDA22, respectively (Fig 1D), were also engineered. However, in contrast to their protein counterparts, which have a flexible linker and trimerization domain at the C-terminus, the DNA immunogens instead contain the native transmembrane and cytoplasmic tail sequences from gp41 so that they can be expressed as membrane-anchored proteins similar to wild-type Env.

### Immunogen characterization

**Fig 2:**
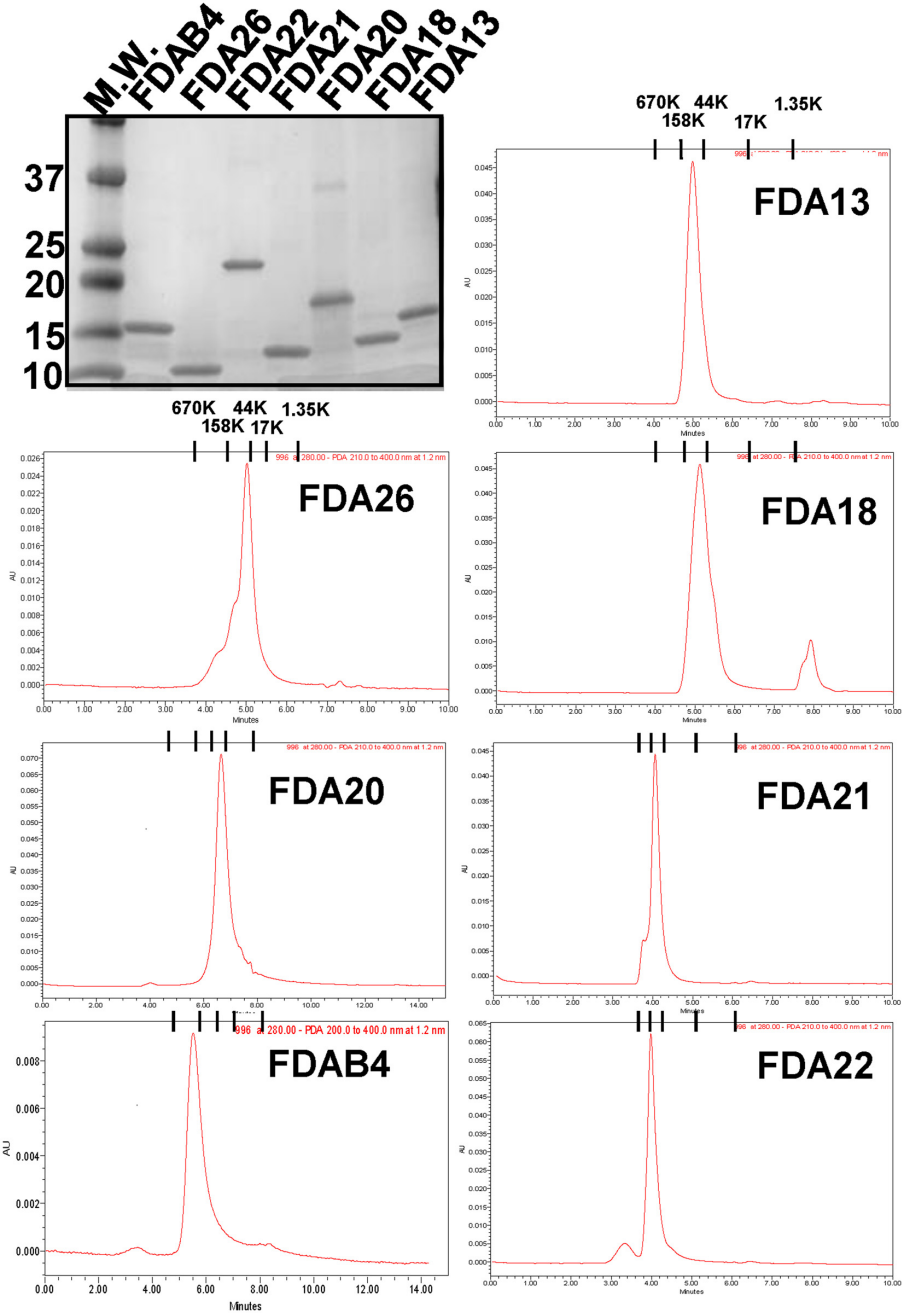
Purity and oligomerization of the gp41 immunogens. (top left panel) SDS-PAGE with Coomassie staining and (remaining panels) size-exclusion chromatography of the gp41 protein immunogens. Molecule mass markers are indicated by bands (M.W. lane) in the gel or tick marks in the chromatograms.

All constructs in Fig 1C were expressed in *E*. *coli*, and the recombinant proteins were isolated from inclusion bodies, purified, and refolded by dialysis. All immunogens were judged to be at least 95% pure by SDS-PAGE and Coomassie blue staining (Fig 2, top left panel). Size exclusion gel chromatography analysis showed that the immunogens eluted as a single major peak at sizes consistent with trimers (Fig 2, remaining panels).

**Fig 3:**
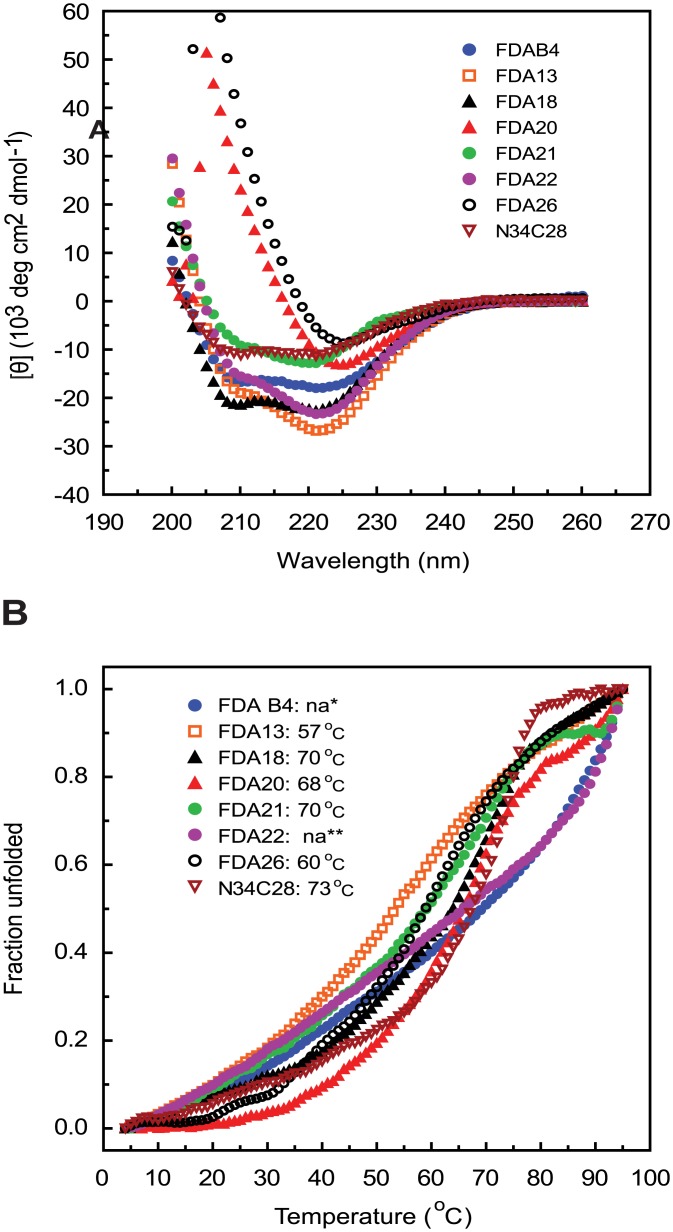
Helical content and thermostability of the gp41 immunogens. (A) Circular dichroism spectroscopy of the gp41 immunogens recorded from 200 to 260 nm. (B) Thermal denaturation curves recording unfolding at 222 nm and the indicated temperatures. Calculated midpoint of thermal transition (Tm) is listed in the legend. “na” means not available due to high thermostability and non-reversible unfolding.

**Fig 4:**
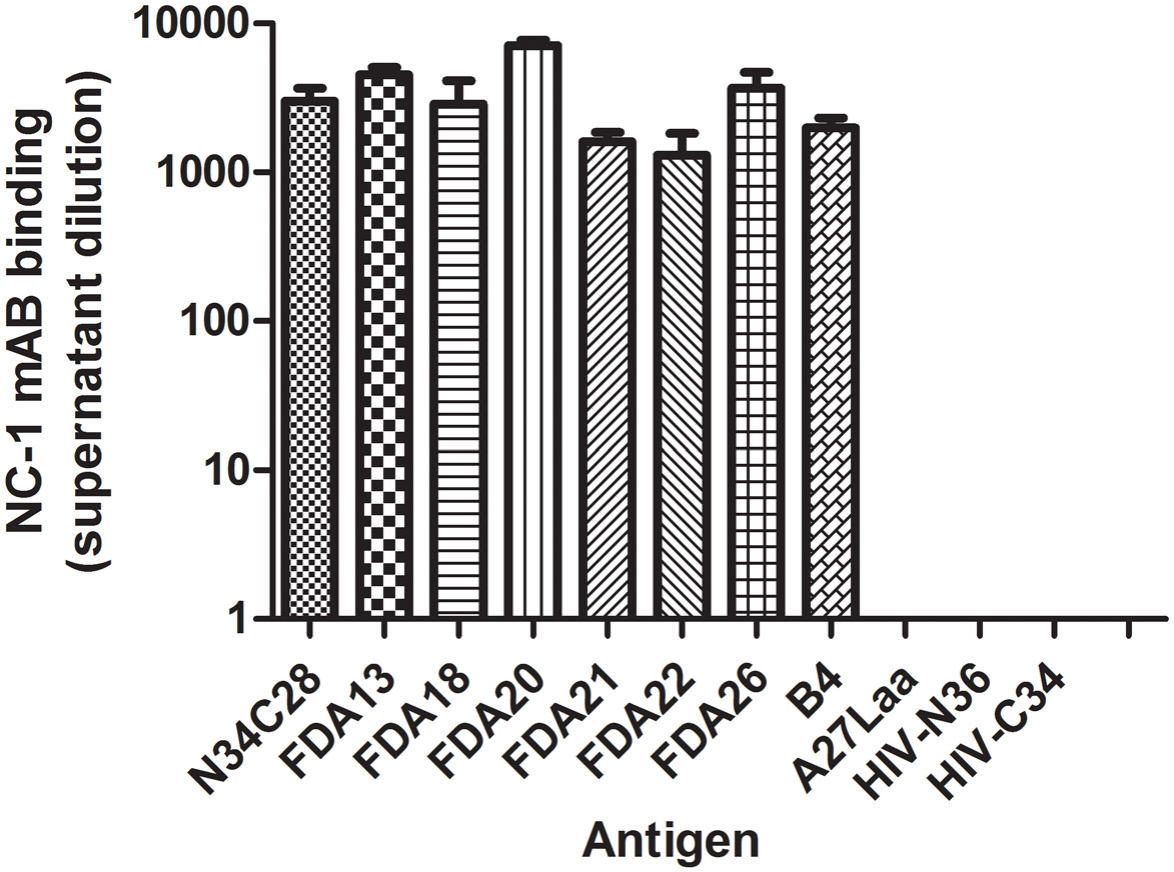
Six-helix bundle structure in the gp41 immunogens. Indirect ELISA of the indicated gp41 immunogens using a monoclonal antibody specific for the six-helix bundle (NC-1)[76]. Positive control is a recombinant six-helix bundle (N34C28). Negative controls are an N-HR peptide (N36), a C-HR peptide (C34) and an irrelevant coiled-coil trimer (A27) which was expressed and purified in a manner similar to the gp41 immunogens [93].

To determine whether the immunogens folded into a 6HB-like structure as designed, the immunogens were also assessed by circular dichroism spectroscopy. All immunogens displayed high helical content (Fig 3A), and thermal denaturation studies demonstrated that they all had high temperatures for their midpoints of transition (Tm) (Fig 3B), consistent with formation of 6HB structures. The presence of a 6HB structure in the immunogens was further assessed using a monoclonal antibody (NC-1) that is specific for the 6HB [76]. These studies showed that the NC-1 monoclonal binds to the immunogens containing both N- and C-heptad repeat domains (FDA13, FDA18, FDA20, FDA21, and FDA26, FDAB4), but not to immunogens that lack both HR1 and HR2 sequences (A27L recombinant protein or N36 or C34 peptides) (Fig 4). Unexpectedly, NC-1 also bound to FDA22, which has the SIV N-HR and C-HR. This reactivity may reflect cross-reactivity of the monoclonals for the SIV 6HB, however, we cannot rule out the possibility that a heterologous 6HB was formed between the SIV N-HR and the HIV C-HR.

The integrity of the 2F5 and 4E10 epitopes in the MPER was evaluated by surface plasmon resonance (SPR). Results showed that all immunogens demonstrated strong binding to 2F5 and 4E10 monoclonal antibodies, with equilibrium dissociation constants (K_D_) in the 10^-7^–10^-10^ M range (Table 1). FDA13 showed a significantly lower K_D_ (~5^-10^) with 2F5, probably due to the presence of an extra 2F5 epitope inserted into the loop region of the immunogen. Insertion of a V3 sequence in the loop, use of the foldon trimerization domain at the C-terminus, use of the SIV 6HB trimerization domain at the N-terminus, or elimination of the C-terminus trimerization domain did not significantly affect antibody binding. Capture ELISA with the 2F5 monoclonal also showed the same pattern of antibody binding to the various immunogens (not shown).

**Fig 5:**
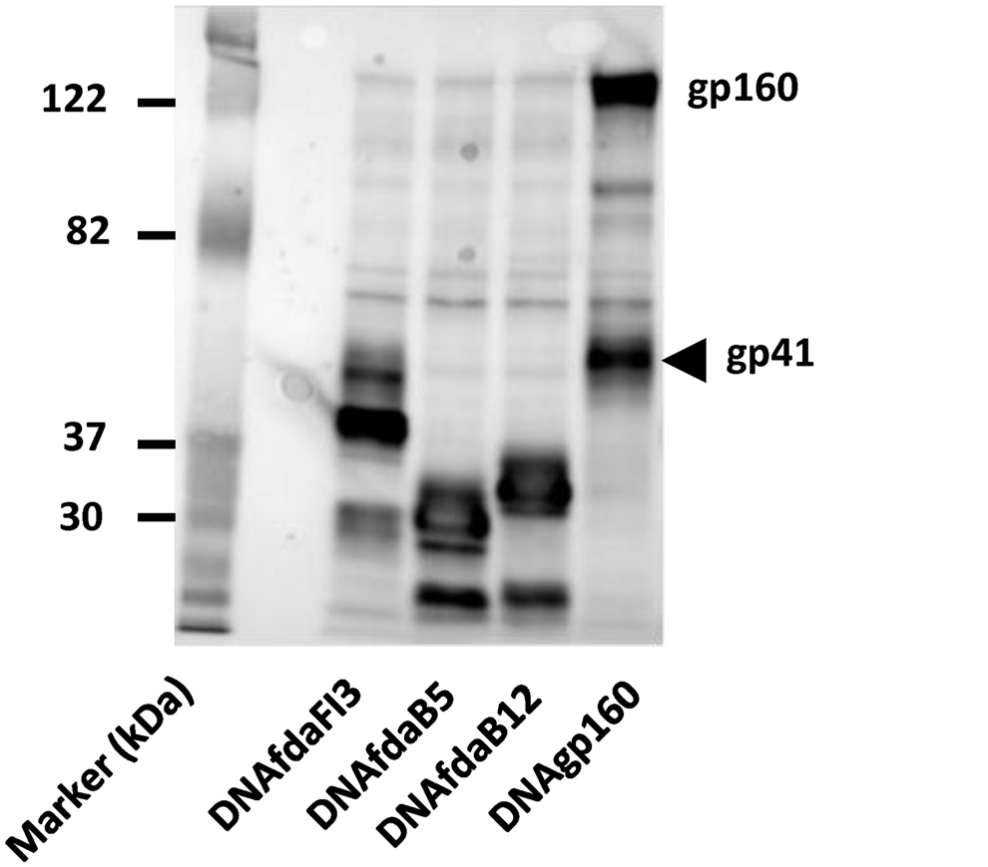
Expression of DNA priming vectors. Immunoblots of cell lysates from 293T cells transfected with the indicated priming vectors and probed with an anti-gp41 antibody (Chessie 8, [77]).

The DNA priming vectors showed good expression of all constructs at the expected size (Fig 5). The expressed membrane-anchored proteins were also all efficiently precipitated by 2F5 and 4E10 (not shown).

### Immunogenicity of oligomeric gp41 immunogens

The immunogens were evaluated for MPER antibody responses in five immunization studies involving different combinations of DNA and protein antigens, as well as immunization protocols. The first study, involving only gp41 protein immunogens and a relatively short immunization schedule, was used to screen the gp41 immunogens with the intent of selecting the best immunogens for additional studies. Four additional studies involving a DNA prime-protein boost protocol and longer immunization schedule where then undertaken in an iterative fashion to address questions raised in the earlier studies.

The soluble gp41 protein immunogens were initially screened for immunogenicity in a protocol involving four 50 μg doses of a protein immunogen, mixed with complete Freund’s adjuvant for the prime and incomplete Freund’s adjuvant for the boosts, and administered subcutaneously in two divided doses at approximately 4 week intervals (Table 2, S1-S7). An additional group received two doses each of the FDA13, FDA18, and FDA22 immunogens given sequentially in various order at four week intervals (Table 2, H1) to see if extra boosting with immunogens sharing the MPER determinant would improve maturation of immune responses to this region.

All immunogens were highly immunogenic and elicited ELISA titers (EU) of at least 10^4^ against the immunizing antigen, with little differences among the immunogens in the different groups (Table 2). To address immunogenicity of MPER epitopes, we evaluated antisera binding to MPER and T20 peptides, which both contain the core 2F5 epitope. T20 is a C-HR peptide that is mostly unstructured in solution, but it can form a helix in the presence of N-HR peptides as it assembles into a 6HB [94]. Overall, we found that the immunogens elicited antibody binding titers to MPER and T20 peptides in the 10^2^–10^3^ EU range, except for the FDAB4 immunogen that elicited lower titers (Table 2). In all cases, titers to T20 were higher than those to the MPER peptide, possibly due in part to antibody binding to the N-terminal part of T20, which is not present in the MPER peptide.

**Fig 6:**
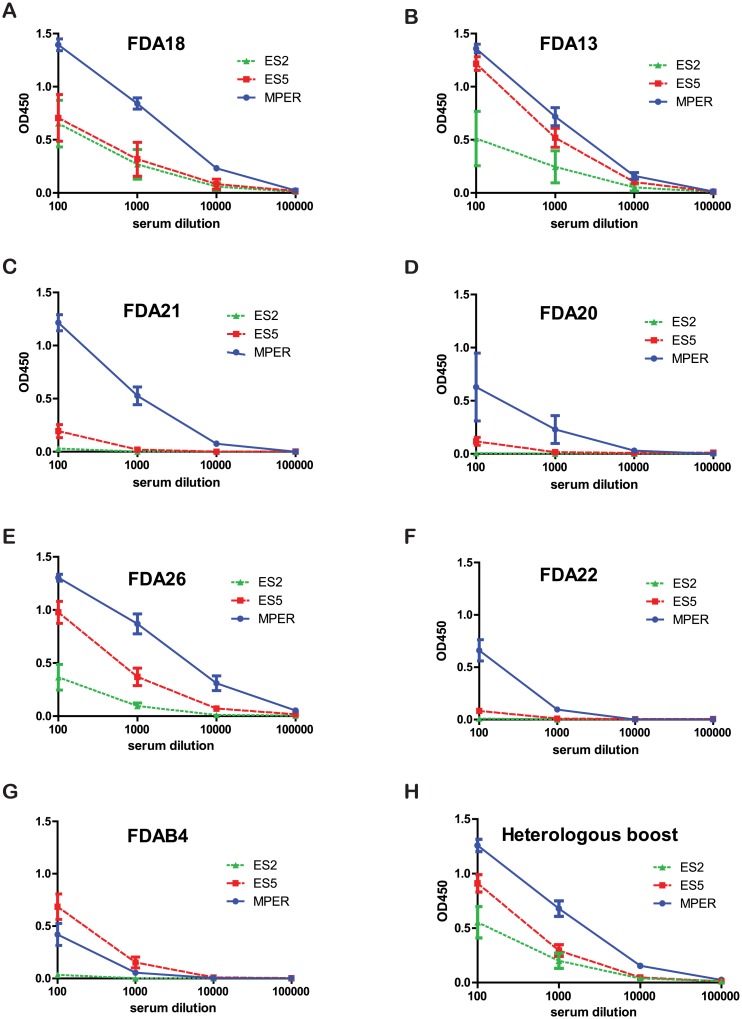
Antisera binding to an MPER peptide or 2F5 epitopes grafted on to protein scaffolds (ES2 and ES5). (A-G) Binding curves of antisera from individual rabbits (identification number listed in legend and color coded for each individual rabbit) immunized with (A) FDA18, (B) FDA13, (C) FDA21, (D) FDA20, (E) FDA26, (F) FDA22, and (G) FDAB4 to an MPER peptide or MPER-scaffolds ES5 or ES2). (H) Binding curves of pooled sera from six rabbits sequentially immunized with two doses each of FDA13, FDA18, and FDA22 immunogens, given in various order as described in Materials and Methods.

When comparing antibody responses elicited by the various immunogens, we found that the FDA13, FDA18, and FDA26 immunogens elicited the highest MPER titers, which were generally at least a log higher than those elicited by the other immunogens. The FDA26 immunogen, which lacked a C-terminal trimerization domain extension, induced the highest antibody titers to the MPER peptide, perhaps due to the availability of more conformations in the relatively unrestrained MPER in this immunogen. The extra MPER epitope in the FDA13 immunogen, however, did not improve MPER titers compared to the FDA18 immunogen, which lacks the extra MPER epitope. The FDA20 immunogen, which contains a V3 immunodominant epitope in the loop region, elicited moderately lower antibody responses to MPER peptides relative to the similar immunogen that lacks the V3 insert (FDA18). Additional studies are needed to determine whether this finding reflects diversion of responses to the dominant epitope in this immunization protocol. Finally, in support of our strategy of using a truncated N-HR to create a short 6HB, we found that the FDAB4 immunogen, which likely forms a more complete (longer) 6HB that extends helical structure into the 2F5 determinant compared to the other immunogens, elicited the lowest antibody titers to both MPER and T20 peptides. In ELISAs on individual rabbit serum (Fig 6 and data not shown), the groups that received FDA26, FDA18, FDA13, FDA21, and heterologous immunogens had higher anti-MPER titers than the group that received the FDAB4 immunogen (p < 0.05, one tailed *t* test). Additionally, when MPER or T20 responses were compared to total antibody responses to the entire immunogen, we noted that among all the immunogens, FDAB4 gave the least MPER focusing, FDA26 gave the best MPER focusing, and the others were more intermediate (Table 2, last two columns).

We further investigated sequential immunizations involving two doses each of FDA18, FDA13, and FDA22 immunogens given in various order to see if MPER responses could be augmented by boosting with different antigens with the shared MPER determinant (Table 2, groups H1). In these groups, responses to the MPER peptide were within the range that was seen for the animals immunized with single antigens. In addition, despite the extended boosting involving a total of six immunizations, compared to the four immunizations used for the single immunogen protocol (groups S1-S7), antibody titers were similar among all the groups and plateaued after the 3^rd^ or 4^th^ immunization (not shown).

Because the MPER peptide is relatively unstructured in the absence of lipid or an antibody binding to it, we also measured antibody binding to two unrelated protein scaffolds with 2F5 epitope grafts, referred to as ES5 and ES2. The former has a more flexible 2F5 epitope, and the latter has a more structured epitope [44]. For most sera, titers to these epitope scaffolds were considerably lower than titers to the MPER peptide, indicating that epitopes in the scaffolds were likely a small subset of those present in the MPER peptide (Fig 6). Overall, titers to the less restrained ES5 scaffold were generally much higher than those to the more restrained ES2 scaffold, a result that mirrors the relative immunogenicity of the epitopes in these scaffolds [44] [95]. Thus our immunogens did not elicit high antibody titers to these more structured 2F5 epitopes.

### Immunogenicity of gp41 oligomeric immunogens in prime-boost studies

We next undertook DNA prime-protein boost immunization studies to evaluate how various combinations of DNA priming vectors and protein boosts affected immunogenicity of the MPER (Table 3). Compared to the immunogenicity studies using only protein immunogens described above, the prime-boost immunization protocol involved longer rests between immunizations and used half the amount of protein immunogen per dose (25 μg), which was mixed with a different adjuvant, MF59, instead of Freund’s adjuvant and delivered intramuscularly at two sites. Priming immunogens were given at weeks 0 and 4, followed by boosting at weeks 12 and 24.

The first study assessed whether DNA priming with a gp160 expression vector affected antibody responses elicited by the FDAB4 gp41 immunogen and how this gp41 immunogen compared to an oligomeric gp140 protein immunogen in eliciting responses to the MPER. The results showed that, in this immunization protocol, the FDAB4 immunogen as a single antigen elicited titers to itself and MPER (Table 3, prime-boost study #1, group N1-2) in a similar range as seen in the earlier immunization protocol (Table 2, group S7). FDAB4 titers were lower in the groups that received the combination of FDAB4 and oligomeric gp140 protein immunogen (Table 3, prime-boost study #1, groups N1-3 and N1-4), likely reflecting the poor immunogenicity of the MPER region in the gp140 immunogen (Table 3, prime-boost study #1, N1-1). All groups, however, had extremely low or undetectable MPER titers, consistent with the poor MPER responses seen with the FDAB4 immunogen in the previous study using the other immunization protocol. Priming with a DNA vector expressing gp160 did not improve MPER responses in boosts with either FDAB4 or gp140 protein immunogens.

The second prime-boost study (Table 3, prime-boost study #2) compared the gp140 protein immunogen to FDA13 and FDA18 immunogens, the latter two immunogens differing only by the FDA13 immunogen having an extra 2F5 epitope in the loop region (Fig 1C). Additionally, two DNA vectors, one expressing gp160 (DNA gp160) and the other expressing the membrane-anchored FDA13 containing the extra 2F5 epitope in the loop along with native transmembrane and cytoplasmic domains (DNAfdaB12, Fig 1D), were assessed as primes for these protein immunogens.

In this study, all groups that received at least two doses of either FDA13 or FDA18 immunogens, including the groups that received priming with either gp160 DNA or gp41 DNA (DNAfdaB12), elicited strong titers to the gp41 immunogen (Table 3, prime-boost study #2, groups N2-1-4), similar to titers seen in the first study using the initial immunization protocol (Table 2). In the groups that received two doses of the FDA13 or FDA18 immunogens followed by boosting with oligomeric gp140 protein, somewhat weaker responses were elicited to the gp41 immunogens, perhaps reflecting the fewer doses of this immunogen. However, antibody responses to the MPER peptide were low in all groups and did not reach statistical differences, though the highest titers were seen in both groups that received the gp41 DNAfdaB12 priming vector followed by gp41 protein boosting with FDA13, which has the extra 2F5 epitope, or FDA18 immunogens (Table 3, prime-boost study #2, groups N2-4 and N2-7).

The third immunization study (Table 3, prime-boost study #3) evaluated two additional gp41 DNA priming vectors, DNAfdaFI3 and DNAfdaB5 (Fig 1D), corresponding to protein immunogens FDA22 and FDA18, respectively, except that the priming vectors contain the HIV Env transmembrane and cytoplasmic for membrane anchoring. These DNA priming vectors were boosted with gp41 immunogens FDA22, FDA18, and FDA20 (Fig 1C), which all share a single MPER determinant near the C-terminal trimerization domain. In this protocol, three additional protein boosts involving an extra dose each of FDA18, FDA22, and FDA20 were given at 4 week intervals following the fourth immunization at 24 weeks in an attempt to further boost MPER responses.

Results from this study again showed strong antibody responses to the gp41 protein immunogen and weak responses to the MPER. Like the prior study, we noted that the groups that received the DNA priming vectors with one of the gp41 constructs gave better MPER responses than the groups that received the gp160 DNA priming vector (Table 3, prime-boost study #3, groups N3-1and N3-2 compared to group N3-3, groups N3-4, and N3-5 compared to N3-6, and groups N3-7 and N-38 compared to N3-9), but the differences failed to reach statistical significance. The highest titers were elicited in the group receiving the DNAfdaFI3 gp41 prime followed by boosting with FDA20 (group N3-7). In this study, the V3 immunodominant epitope in the loop region of FDA20 did not appear to impair responses to the MPER. Compared to the bleeds at week 24 (not shown), the extension involving three additional protein boosts gave no improvement in antibody responses to the MPER.

In the fourth prime-boost immunization study (Table 3, prime-boost study #4), we combined DNAfdaB12, which is the gp41 DNA priming vector with the extra 2F5 epitope, with the FDA26 immunogen, which gave the best MPER responses in the initial immunogenicity study. In this case, we used both protein and DNA for the priming, delivering each immunogen at separate sites, followed by protein boosting (Table 3, prime-boost study #4, N4-1). As seen in the prior prime-boost studies, responses were strong to the protein immunogen, but weak to the MPER. Although the differences failed to reach statistical significance, gp41 DNA priming again tended to elicit slightly higher MPER responses compared to protein alone. Curiously, FDA26 elicited very low titers to the MPER in this study, as did FDA13 and FDA18 in the second and third prime-boost studies above, compared to the initial immunogenicity study using only protein immunogens. This finding raises the possibility that protocol attributes, such as choice of adjuvant, may differentially affect responses to the MPER epitope.

### Neutralization studies

**Fig 7:**
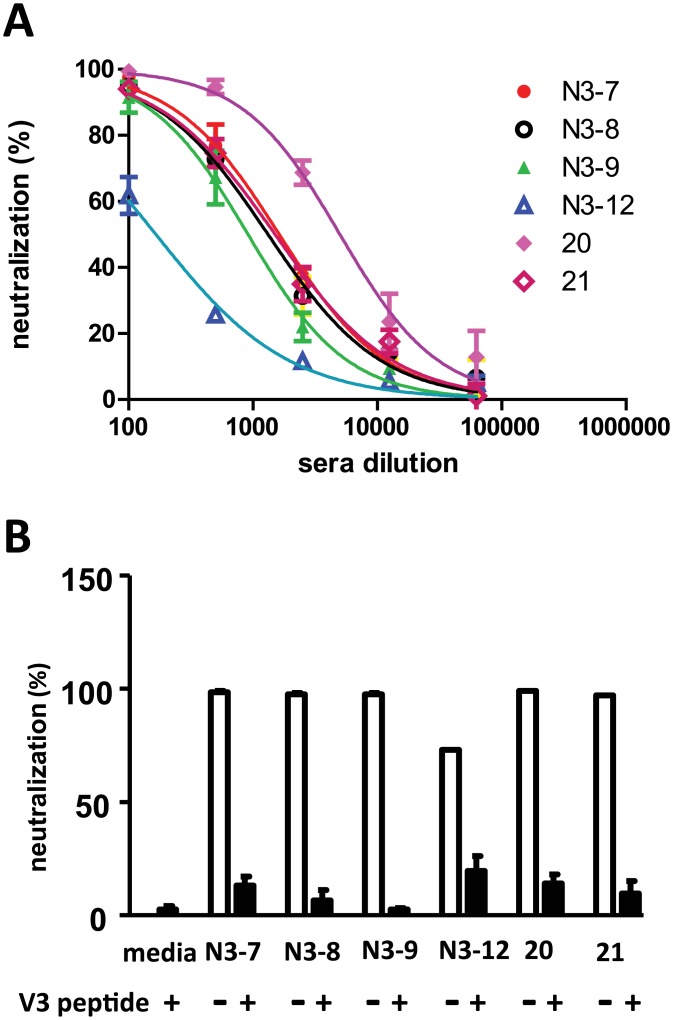
Neutralization of SF162 pseudoviruses by sera from rabbits immunized with the FDA20 immunogen. (A) Dose-response neutralization curves of sera from two rabbits from the first immunization study (numbered 20 and 21) and pooled sera from rabbit groups from the third prime-boost study (N3-7, N3-8, N3-9, N3-12). (B) Neutralization is abrogated by addition of a V3 peptide matching the sequence in the FDA20 immunogen.

All sera were assessed for neutralization activity against a small panel of pseudoviruses that included mostly neutralization-sensitive Envs, as well as a few Tier 2 Envs (see Materials and Methods for details). Across all studies involving only gp41 immunogens, only rabbits immunized with the gp41 immunogen containing the V3 domain (clade C) inserted in the loop of the gp41 trimer (FDA20) elicited potent neutralization, which was strain specific. Among the strains assessed, high neutralization titers were achieved only against the SF162 strain (Fig 7A and data not shown). Lack of neutralization to pseudoviruses with the other Envs likely reflects sequence differences in the V3 region. Groups that received gp140 immunogens showed modest neutralization titers to a few Tier one isolates (data not shown), consistent with prior studies using this gp140 immunogen [82].

We further determined that the neutralization activity in groups immunized with the FDA20 immunogen mapped to the V3 region by adsorbing sera with a V3 peptide corresponding to the sequence used in the gp41 immunogen (Fig 7B). In remaining constructs lacking the V3 insert, there was only sporadic weak neutralization against one or two neutralization sensitive strains (data not shown). Therefore, although most of the trimeric gp41 immunogens elicited moderate MPER peptide binding antibody titers, these antibodies failed to neutralize. This finding suggests that our constructs failed to elicit the type of antibodies that can reach and bind neutralizing determinants with high enough affinity or that such antibodies were present only at very low titers.

## Discussion

Generating immunogens that elicit robust neutralizing antibody responses to the MPER remains a high priority despite many failed attempts using a variety of strategies [96]. In this study, we used segments of the N- and C-HR domains of gp41 to create soluble, trimeric immunogens to model a fusion-intermediate conformation of Env that presents the MPER unhindered by gp120. Several variations of the gp41 immunogen were evaluated in protein only and prime-boost immunization protocols to evaluate how extra epitopes, 6HB bundle length, and different immunization protocols affect antibody responses to the MPER.

Overall, the best gp41 immunogens and immunization protocol elicited moderate antibody binding titers to the MPER, but these sera lacked significant neutralizing activity to this determinant. Extra boosting with heterologous immunogens that share the MPER determinant did not increase MPER titers or neutralization activity. Variations in the immunogens and immunization protocols, however, appeared to influence antibody responses to this region.

In the first immunization protocol involving only protein immunogens (Table 2), the immunogens with a truncated N-HR designed to form a short 6HB (Fig 1, FDA13, FDA18, FDA20, FDA21, and FDA26) elicited higher MPER binding antibody titers than the immunogen with the longer N-HR segment that is expected to form a longer 6HB (FDAB4). These data suggest that shortening the 6HB to mitigate extension of helical structure from the C-terminal end of the C-HR into the MPER can improve antibody responses to MPER epitopes. Importantly, the immunogens with the shorter N-HR still retained the desirable features of the trimeric 6HB that confer stability and multi-valency to the immunogen (Figs 3 and 4). Indeed, insertion of a V3 determinant in the loop connecting N-HR and C-HR in the gp41 scaffold elicited potent, type-specific neutralizing antibodies to this determinant (Fig 7), showing that the gp41 trimer can be a useful platform for epitope presentation.

However, insertion of an extra MPER determinant in the loop (FDA13), resulting in six MPERs per trimer, did not improve antibody responses to this region. The inability to boost MPER titers with extra epitopes may be due to the inherently poor immunogenicity of the MPER, perhaps relating in part to self-tolerance to epitopes in this region. In this case, alternative approaches, such as use of specialized adjuvants or immunization schedules, might be needed to augment immunogenicity of self epitopes. Use of an unstructured peptide to detect MPER antibodies may also limit detection of antibody responses to structured determinants in the native Env. In this regard, we evaluated ELISA titers in the sera to two additional antigens with structured 2F5 epitopes grafted onto protein scaffolds. We found that titers to the epitopes in the protein scaffolds were generally much lower compared to titers to the MPER peptide (Fig 5). This result indicates that the structured epitopes presented in the scaffold are represented to some degree in the MPER peptide, but that our immunogens did not elicit high titers to these structured epitopes.

Because a membrane environment may be important for stabilizing MPER structure and antibody binding [35,58,97,98], we generated three membrane-anchored gp41 constructs with the native transmembrane and cytoplasmic tail domains for use as DNA priming vectors (Fig 1D). DNA priming can also improve responses to protein boosts [99], but comparisons of gp160 and gp41 fusion-intermediate priming vectors has not been previously reported. In the prime-boost studies (Table 3), titers to the entire immunogen were high (~10^4^ endpoint dilution) and similar to those seen in the analogous groups in the initial immunization studies involving only protein immunogens (Table 2). Curiously, antibody titers to the MPER were much lower than the MPER titers observed in the initial immunization study involving only the protein immunogens.

Across all prime-boost studies, the highest MPER titers (~10^2^ EU) were seen in groups that received a gp41 DNA prime followed by gp41 protein (Table 3, prime-boost #2, groups N2-4 and N2-7, and Table 3, prime-boost #3, groups N3-7). This finding was observed in two independent studies involving three different groups of animals and two different gp41 DNA primes (DNAfdaB12 and DNAfdaFI3), followed by one of three different gp41 protein boosts (FDA13, FDA18, FDA20). In particular, the gp41 DNA primes appeared to compare favorably to gp160 DNA primes or gp140 protein boosts in eliciting MPER titers, indicating that the gp41 immunogens may better focus immune response towards MPER epitopes compared to gp160 or gp140. Additionally, in the prime-boost protocol, gp41 DNA priming tended to be better than protein immunogens alone in eliciting MPER titers. While the MPER titers are low, and the differences don’t reach statistical significance between the groups, these consistent findings suggest that the membrane-anchored gp41 construct was helpful in eliciting antibodies to the MPER.

Why the groups that received only the protein immunogens in the prime-boost studies had lower titers to the MPER peptide, but not to the immunogen itself, compared to the analogous groups in the initial immunogenicity study is unclear, but differences in the immunization protocols may have played a role. The initial immunization protocol involving only protein immunogens used twice the amount of protein per dose (50 μg) along with Freund’s adjuvant, which was delivered subcutaneously at four week intervals. In the prime-boost protocol, doses (25 μg) were delivered intramuscularly at weeks 0, 8, 12 and 24 with the MF59 adjuvant. Significantly, the prime-boost protocol efficiently elicited high antibody titers to the immunogen itself, even in groups that only received two doses of the protein immunogen, yet MPER titers were extremely low. This finding raises the possibility that specific factors employed in the immunization protocol, such as choice of adjuvant or mode of administration, may differentially affect MPER immunogenicity compared to other epitopes. Further studies are needed to address this issue.

In summary, these comparative studies using gp41 immunogens based on the trimerization properties of the N- and C-HR indicate that mitigating extension of helical structure into the MPER region can improve antibody titers to this region. Priming vectors with similar gp41 constructs also appear better than gp160 in eliciting MPER antibodies. Differences in the immunization protocols or adjuvant possibly also alter antibody responses to MPER relative to other epitopes. Nonetheless, while better immune focusing on the MPER is a critical first step in eliciting neutralizing antibodies to this region, further work is needed to understand how to elicit high titers of the right kind of antibodies that have high affinity and are able to reach the epitope at the right time to achieve neutralization.

## Supporting Information

S1 Table Amino acid sequence of immunogens. (TIF)
