# Supplementary figures and images for: Immunogens Modeling a Fusion-Intermediate Conformation of gp41 Elicit Antibodies to the Membrane Proximal External Region of the HIV Envelope Glycoprotein
Source: PLoS One. 2015 Jun 18;10(6):e0128562. doi: 10.1371/journal.pone.0128562 (PMC4472232; doi:10.1371/journal.pone.0128562)

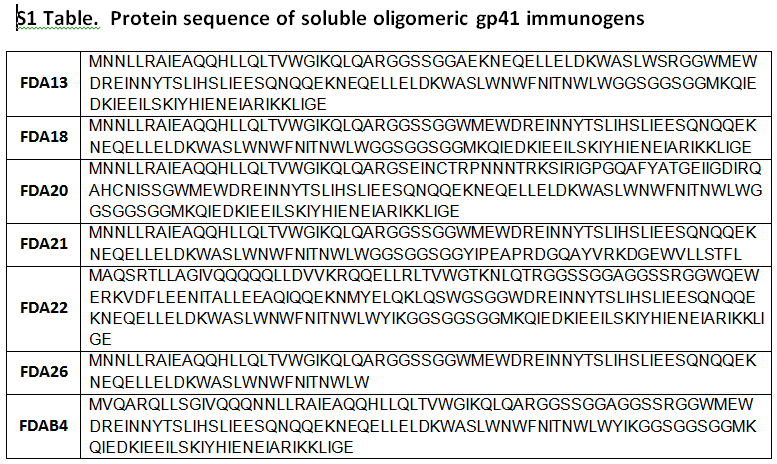

Supplement: S1 Table — (TIF) [file pone.0128562.s001.tif]
